# Association between women’s perceived ideal gestational weight gain during pregnancy and pregnancy outcomes

**DOI:** 10.1038/s41598-018-29936-z

**Published:** 2018-08-01

**Authors:** Kohei Ogawa, Naho Morisaki, Haruhiko Sago, Takeo Fujiwara, Reiko Horikawa

**Affiliations:** 1 Center of Maternal-Fetal, Neonatal and Reproductive Medicine, National Center for Child Health and Development, Tokyo, Japan; 2 Department of Social Medicine, National Center for Child Health and Development, Tokyo, Japan; 3 Collaborative Departments of Advanced Pediatric Medicine, Graduate School of Medicine, Tohoku University, Sendai, Japan; 4 Department of Global Health Promotion, Tokyo Medical and Dental University, Tokyo, Japan; 5 Department of Endocrinology, National Center for Child Health and Development, Tokyo, Japan

## Abstract

We aimed to clarify which beliefs motivate women to control their weight during pregnancy and how such values influence pregnancy outcomes. Using a questionnaire administered during mid- to late- pregnancy in a hospital-based prospective cohort study, we explored women’s perceived ideal GWG and their reasons for having this ideal. Using multivariate regression, we evaluated the association between women’s perceived ideal GWG and pregnancy outcomes. Among 1,691 normal and underweight women, the most common reason women thought avoiding excessive weight gain was important was “for ease of delivery and/or her health and well-being”. 912 (54%) women wished to maintain their GWG below 12 kg, the upper limit recommended by the Japanese governmental guidelines, and had a lower actual GWG compared to those who had less stringent notions of GWG. Compared to women whose perceived ideal GWG was 12 kg, those who considered their perceived limit to be lower had infants with lower birthweight on average despite no significant reduction in cesarean delivery rate or post-partum body weight retention. Our findings suggest that women who believe they should limit their weight gain to an amount lower than the upper limit of current guidelines succeeded in gaining significantly less weight but received no additional benefit.

## Introduction

Guidelines on weight gain during pregnancy are based upon robust evidence showing that gestational weight gain (GWG) influences maternal and child health^1^. Despite the current recommendations on GWG based on scientific evidence from multiple countries^2,3^, previous studies have demonstrated that a significant proportion of women end up not meeting the recommendations^4^ and that such women are at a higher risk of adverse pregnancy outcomes^5^. As many as 68% of women in the United States^6^ and 46% of non-obese women in Japan (Japan does not have documented recommendations for obese women) reported experiencing weight gain exceeding the range recommended by the respective guidelines^2,3^.

Many educational interventions have aimed to steer GWG towards a favorable range; unfortunately, only a few have succeeded^7,8^. One possible explanation for this failure may lie in the fact that interventions have not attempted to account for women’s underlying values related to gaining weight. How important a person considers the consequence of their actions is likely to influence their motivation to act, as can be seen from studies analyzing success in smoking cessation^9^. For instance even when provided with the same information regarding recommendations for gestational weight gain, the women’s personal views and values may have a substantial influence on whether and how she decides to follow such recommendations. Thus, understanding what women consider as appropriate GWG and to identifying the values driving this perception are important for designing effective educational interventions to improve actual GWG. We found one study investigating the association between women’s perceived ideal GWG and actual GWG^10^. However, it did not investigate the GWG considered by women to be optimal nor how women’s underlying beliefs might affect their behavior and pregnancy outcomes. Thus in this prospective cohort study, we investigated the factors related to how women determine their perceived ideal GWG as well as how such ideals affect pregnancy outcomes, using normal or underweight women as subjects.

## Material and Methods

### Study sample

Our study was based on a prospective, hospital-based birth cohort^11,12^. Pregnant women making antenatal visits to the National Center for Child Health and Development (NCCHD), a tertiary hospital in Tokyo managing approximately 1,500 deliveries annually, between May 13, 2010 and November 28, 2013 were recruited at their first antenatal visit, which usually takes place during weeks 6–14 of gestation. The participants were asked to fill out multiple questionnaires, including one self-administered questionnaire on demographics at recruitment; a second questionnaire on the respondent’s perceptions of ideal GWG and her reasons for having these perceptions was given to the women by researchers during a mid- to late-pregnancy visit and returned by the respondent at a later visit. At NCCHD, a booklet that includes information on the Ministry of Health, Labour and Welfare GWG pregnancy guidelines is handed out at the first trimester antenatal visit, and at each antenatal visit thereafter midwives monitor the women’s current weight gain and provide advice if the gain is too rapid or too slow in order to help prospective mothers maintain their GWG within the range recommended by the government guidelines.

Out of 4,164 subjects recruited, 2,310 (55%) consented to participate. We excluded 84 women with multiple pregnancies, 133 women with a pre-pregnancy BMI over 25 kg/m^2^, and 96 women with diabetes mellitus or gestational diabetes, as there is no consensus or guideline on GWG recommendations for multiple pregnancies or overweight women with a BMI exceeding 25 kg/m^2^ in Japan^13^, and women with a diagnosis of diabetes are likely to receive guidance from a nutritionist on weight gain, which may influence their perception of appropriate weight gain during pregnancy. In total 1,691 (85%) of the remaining 1,987 women returned both questionnaires with complete information on baseline demographics. All the women were followed until delivery and were included in our final sample. At one month after delivery, the women were asked whether they would like to continue participating in the cohort by answering additional questionnaires and attending visits, and 1,385 (82%) agreed. At 12 months post-partum, a questionnaire including questions about current weight were administered and completed by 1,032 (75%) of the subjects.

This study protocol was approved by the Institutional Review Board at the NCCHD on August 2, 2010 (project number 417), and written informed consent was obtained from each participant. All methods were performed in accordance with the relevant guidelines and regulations.

### Measures

#### Women’s perceived ideal GWG and their reasons for avoiding excessive weight gain

In a questionnaire administered during mid to late pregnancy (ranging from 26 to 38 weeks), we asked women how much GWG they thought was appropriate for themselves (their perceived ideal GWG) and used two questions to determine whether they thought avoiding excessive weight gain was important and if so, why.

The first question was, “How much weight do you think is appropriate for yourself to gain during pregnancy?” to which the respondent was allowed to answer with, “I do not know” or by indicating an upper limit or a range (upper and lower limits) in kilograms. As the upper limit of GWG recommended by the Ministry of Health, Labour and Welfare guidelines is 12 kg for all women with a BMI 25 kg/m^2^ or lower (i.e., the recommended range is 7–12 kg for women with BMI below 18.5 kg/m^2^ and 9–12 kg for women with a BMI 18.5–25 kg/m^2^), we categorized women according to the upper limit of their perceived ideal GWG into five categories: “higher than the guideline recommendation (12 kg<)”, “same as the guideline recommendation (12 kg)”, “within the recommended range but lower than the upper limit,” “lower than the guideline recommendation,” and “no limit/not sure.”

The second question was, “Do you think it is important not to gain too much weight during pregnancy?” Possible responses were “very much so,” “somewhat so,” “not sure,” “not much,” or “not at all.”

Women who answered the second question with “very much so” or “somewhat so” were asked to answer a third question, “Why do you think so?” Possible responses (multiple answers were allowed) were, “to bear a healthy child,” “for ease of delivery,” “to get my body back into shape quickly after delivery,” “to avoid stretch marks,” “because my doctor/nurse/midwife said so,” “because my family/friends said so,” “no particular reason,” and “to avoid lifestyle-related diseases later in life.” Responses to questions 2 and 3 were combined to create the following four categories: “women who think they should avoid excessive weight gain to have a healthy child”(those who answered “very much so” or “somewhat so” to question 2 and “to have a healthy child” to question 3), “women who think they should avoid excessive weight gain for ease of delivery and/or health”, (those who answered “very much so” or “somewhat so” to question 2 and responded “for ease of delivery”, “to get my post-partum body back into shape quickly after delivery,” “to avoid stretch marks” or “to avoid lifestyle-related diseases later in life” to question 3), “women who think they should avoid excessive weight gain because they were told to do so” (those who answered “very much so” or “somewhat so” to question 2 and “because my doctor/nurse/midwife said so” or “because my family/friends said so” to question 3) and “women who think they should avoid excessive weight gain for no reason in particular” (those who answered “very much so” or “somewhat so” to question 2 and responded “for no reason in particular” or gave no response to question 3). The original questions (translated into English) are shown in Appendix Fig. 1.

#### Demographic data

Maternal sociodemographic data were collected from the questionnaire responses and categorized as annual household income (<4 million yen, 4–8 million yen, >8 million yen, or no answer), and maternal education (university graduate, community college graduate, or school or training school education only).

Trained research staff collected the following baseline characteristics from the medical charts kept by obstetricians and midwives: maternal age (15–29, 30–34, 35–39, 40–49 years), parity (0, 1, or more), history of previous preterm delivery (yes/no), and smoking during pregnancy (yes/no/missing). Self-reported pre-pregnancy height and weight as well as weight measurements at each antenatal visit (average: eight visits) were also retrieved from the medical charts. Pre-pregnancy BMI was calculated and categorized as under 18.5 kg/m^2^, 18.5 to 25 kg/m^2^, or 25 kg/m^2^ or more, according to the categorization used in the Japanese national guidelines on GWG recommendations^14^. GWG was calculated by subtracting the self-reported pre-pregnancy weight from weight at birth measured on either the day they were admitted for delivery on the day of delivery if they were admitted beforehand, and substituted by measurement at the last antenatal visit if these were not available (range 28–42 weeks). GWG was adjusted for gestational week at delivery to calculate weight gain at 40 weeks, using a methodology previously reported which assumed linear increase in GWG in the third trimester^15^. Post-partum weight retention at 12 months (PPWR) was calculated using weight at birth and self-reported weight at 12 months post-partum as well.

Data on the pregnancy outcomes were also obtained from the medical charts. Birth weight z-scores were calculated for each day of gestation using the Japanese birth weight curves as a ref.^16^, and birth weight below the 10th percentile of the general population (stratified by parity and sex of the infant) was defined as small for gestational age (SGA) using the same reference. Low birth weight was defined as birthweight below 2500 grams. Gestational length was based on the best obstetric estimate, and preterm delivery was defined as delivery before 37 completed weeks of gestation.

#### Statistical analysis

First, we compared maternal and infant demographics, and determined whether the respondents thought they should avoid excessive weight gain and why they thought so by using the upper limit of women’s perceived ideal GWG ranges in a test for trend analysis. Next, to estimate the association between the upper limit of perceived ideal GWG with pregnancy outcomes (GWG, birth weight, birth weight z-score, gestational length, and PPWR) and the risk of adverse outcomes (low birth weight, SGA, preterm delivery, and cesarean delivery), we conducted linear regression analysis for continuous outcomes, and logistic regression analysis for binary outcomes. For these analyses, we constructed crude and multivariate regression models after adjusting for height and BMI (included as continuous variables), maternal age, parity, history of previous preterm delivery, maternal education, family income, and sex of the infant (included as categorical variables), as previous studies suggested that these characteristics were strongly associated with birth outcomes and might be associated with GWG as well. For the analyses, we used the upper limit of GWG recommended by Ministry of Health, Labour and Welfare guidelines (i.e., 12 kg) as the reference point.

For sensitivity analysis, we adjusted for the reported lower limit of perceived ideal GWG on a sub-group consisting of 1,240 women who had reported both the upper and lower limits of their perceived ideal range. For this analysis we additionally included the width of the reported range (upper limit – lower limit) as a co-variate in the model as previously described. We chose this method rather than including the reported lower limit directly because the upper and lower limits of the perceived ideal weight gain ranges were strongly correlated (Appendix Fig. 2).

All descriptive and statistical analyses were performed using STATA version 13 (STATA Corp, College Station, TX). Statistical significance was set at 0.05, and all statistical tests were two-tailed.

### Data availability

The datasets generated during and/or analyzed during the current study are available from the corresponding author on reasonable request.

## Results

Among 1,691 women, 419 (25%) women stated that 12 kg (which is the same as the government guidelines) was the upper limit of their perceived ideal GWG range. In addition, 912 women (54%) reported the upper limit of their perceived ideal GWG range to be below 12 kg. Among these women, 879 (52%) women reported an upper limit within the guideline ranges, and the remaining 33 (2%) women reported an upper limit below the guidelines. Furthermore, 112 (7%) women selected a perceived ideal range with an upper limit higher than that of the guidelines (12 kg<), and 248 (15%) responded with “not sure/no answer.” The range and interquartile ranges (IQR) for the upper limit, lower limit, and range of perceived ideal GWG were as follows: upper limit [median 10 kg, IQR 10–12 kg, range 2–20 kg], lower limit [median 8 kg, IQR 7–8 kg, range 0–14 kg], range [median 3 kg, IQR 2–4 kg, range 1–10 kg].

Table 1 shows the maternal and infant characteristics according to the women’s perceived ideal GWG categories with reference to the Ministry of Health, Labour and Welfare guidelines. Women whose perceived ideal GWG was lower than the upper limit in the current Japanese recommendations were older, more likely to be multi-para, had a higher BMI (although all women were normal or underweight), had less income, and were less likely to have graduated college. On the other hand, maternal height, smoking status, sex of the infant, or experience of previous preterm delivery did not differ between categories. Women with lower perceived ideal GWG categories had a lower GWG (observed or expected weight gain at 40 weeks), lower birth weight, lower birth weight z-score, and lower gestational age at delivery. They also had a higher rate of preterm delivery and less PPWR, but the rates of SGA and cesarean delivery did not show a significant trend.

**Table 1:** Maternal and infant characteristics among 1,691 women.

		Women’s perceived ideal GWG categorized according to the Ministry of Health, Labour and Welfare guidelines										
		Lower than guideline range (n = 33)		Within guideline range (n = 879)		Same as guideline upper limit (n = 419)		Above guideline range (n = 112)		Not sure/no answer (n = 248)		p-value for trend
Age (yrs), mean (SD)		35.5	(4.2)	36.0	(4.3)	35.4	(4.1)	35.1	(4.2)	36.1	(4.1)	P = 0.012
Height (cm), mean (SD)		158.6	(6.2)	159.5	(5.4)	159.4	(5.2)	160.0	(5.5)	159.4	(5.9)	P = 0.362
BMI (kg/m^2^), mean (SD)		18.5	(1.7)	20.1	(1.9)	19.7	(1.7)	19.9	(1.9)	20.1	(2.0)	P = 0.165
Highest education	University, n (%)	17	(52%)	534	(61%)	306	(73%)	71	(63%)	139	(56%)	P = 0.010
	Community college, n (%)	10	(30%)	169	(19%)	58	(14%)	19	(17%)	54	(22%)	
	High school, training school, n (%)	6	(18%)	176	(20%)	55	(13%)	22	(20%)	55	(22%)	
Annual income	Over 8 million yen, n (%)	19	(58%)	465	(53%)	253	(60%)	70	(63%)	128	(52%)	P = 0.030
	4–8 million yen, n (%)	8	(24%)	329	(37%)	116	(28%)	25	(22%)	87	(35%)	
	Under 4 million yen, n (%)	2	(6%)	50	(6%)	26	(6%)	13	(12%)	23	(9%)	
	Missing, n (%)	4	(12%)	35	(4%)	24	(6%)	4	(4%)	10	(4%)	
BMI	18.5–25 (kg/m^2^), n (%)	24	(73%)	175	(20%)	104	(25%)	29	(26%)	54	(22%)	P = 0.001
	−18.5 (kg/m^2^), n (%)	9	(27%)	704	(80%)	315	(75%)	83	(74%)	194	(78%)	
Primiparity, n (%)		14	(42%)	495	(56%)	296	(71%)	57	(51%)	154	(62%)	P = 0.011
Previous preterm delivery, n (%)		0	(0%)	43	(5%)	11	(3%)	5	(5%)	7	(3%)	P = 0.433
Smoking during pregnancy, n (%)		1	(3%)	14	(2%)	5	(1%)	6	(5%)	7	(3%)	P = 0.140
Male infant, n (%)		20	(61%)	468	(53%)	215	(51%)	56	(50%)	121	(49%)	P = 0.274
Small for gestational age, n (%)		5	(15%)	60	(7%)	23	(6%)	7	(6%)	26	(11%)	P = 0.195
Birth weight <2500 g, n (%)		5	(15%)	86	(10%)	28	(7%)	7	(6%)	22	(9%)	P = 0.030
Preterm delivery, n (%)		1	(3%)	49	(6%)	13	(3%)	2	(2%)	9	(4%)	P = 0.024
Preeclampsia, n (%)		2	(6%)	21	(2%)	8	(2%)	1	(1%)	8	(3%)	P = 0.140
Cesarean delivery, n (%)		8	(24%)	221	(25%)	111	(27%)	33	(30%)	75	(30%)	P = 0.301
Weight gain at 40 weeks^+^(kg), mean (SD)		8.2	(3)	10	(3.3)	10.9	(3.2)	11.8	(3.5)	10.3	(3.8)	P = 0.001
Birthweight (grams), mean (SD)		2,826	(332)	2,993	(418)	3,040	(401)	3,050	(402)	3,005	(383)	P = 0.005
Gestational age (weeks), mean (SD)		38.9	(1.4)	38.7	(1.5)	38.9	(1.4)	38.9	(1.2)	38.9	(1.3)	P = 0.023
Birthweight z-score, mean (SD)		−0.6	(1.0)	0.1	(1.0)	0.1	(1.0)	0.1	(1.1)	0.1	(1.0)	P = 0.009
PPWR^a^ at 12 months (kg), mean (SD)		−0.1	(2.1)	−0.8	(3.0)	−0.4	(2.8)	0.1	(3.1)	0.0	(3.8)	P = 0.022^b^

^a^PPWR: post-partum weight retention. Analysis for this cell is n = 1,032.

^b^P for trend for missing PPWR value by the category of the upper limit of women’s upper limit of perceived ideal GWG p = 0.42.

^c+^Weight gain at 40 weeks was calculated from all antenatal weight gain measurements available assuming linear increase in gestational weight in the third trimester.

Table 2 shows by how much the respondents thought they should avoid excessive weight gain and why they thought so. Of the 1,691 subjects, 1,569 (93%) answered that they considered not gaining too much weight during pregnancy to be “very much” or “somewhat” important. Some 1,395 (83%) women answered that weight should be controlled for ease of delivery and/or maternal health while 1,108 (66%) answered that weight should be controlled in order to bear a healthy child, and 496 (30%) answered that weight control during pregnancy was important based on advice from health professionals or others. Only nine (<1%) women did not give a reason or answered, “no reason in particular.”

**Table 2:** Reasons associated with the range of perceived ideal gestational weight gain among 1,691 women.

	Women’s upper limit of perceived ideal GWG categorized according to the Ministry of Health, Labour and Welfare guidelines										
	Lower than guideline range (n = 33)		Within guideline range (n = 879)		Same as guideline upper limit (n = 419)		Above guideline range (n = 112)		Not sure/no answer (n = 248)		
Reported upper limit of gain (kg), mean (SD)	7.2	(1.5)	9.7	(0.7)	12.0	(0)	14.3	(1.1)	NA		
**Does the woman think it is important to not gain too much weight during pregnancy?**											
											p-value for trend*
Very much so, n (%)	19	(57%)	402	(51%)	157	(37%)	25	(22%)	51	(21%)	p < 0.001
Somewhat so, n (%)	11	(33%)	445	(46%)	232	(55%)	72	(64%)	155	(63%)	
Not sure/not much/not at all, n (%)	3	(9%)	31	(4%)	157	(7%)	15	(13%)	42	(17%)	
No answer, n (%)	0	(0%)	1	(0%)	0	(0%)	0	(0%)	0	(0%)	
**Whether the woman thinks she should avoid excexxive GWG weight for the following reasons:**											
											**p-value for trend**
To bear a healthy child, n (%)	23	(70%)	631	(70%)	312	(75%)	74	(66%)	139	(56%)	p = 0.816
For easy delivery and/or her health, n (%)	28	(85%)	779	(89%)	359	(86%)	91	(81%)	177	(71%)	p = 0.031
Because she was told to do so, n (%)	9	(27%)	284	(32%)	126	(30%)	40	(36%)	77	(31%)	p = 0.770
For no reason in particular, n (%)	0	(0%)	3	(0%)	2	(1%)	0	(0%)	4	(2%)	p = 0.888

*Analysis conducted by assuming both factors as ordinal variables.

Women whose perceived ideal GWG were lower were more likely to answer that they thought avoiding excessive weight gain was “very much” important, and less likely to answer “somewhat” important or “not sure/not much/not at all” (p for trend <0.001). These women were also more likely to answer that avoiding excessive weight gain was important “for ease of delivery and/or her health” (p = 0.031). However, the proportion of women who thought it was important to avoid excessive weight gain “to bear a healthy child” or “because (they were) told to do so” did not differ in terms of the chosen limit (p = 0.816, p = 0.770 and p = 0.888, respectively).

**Figure 1:**
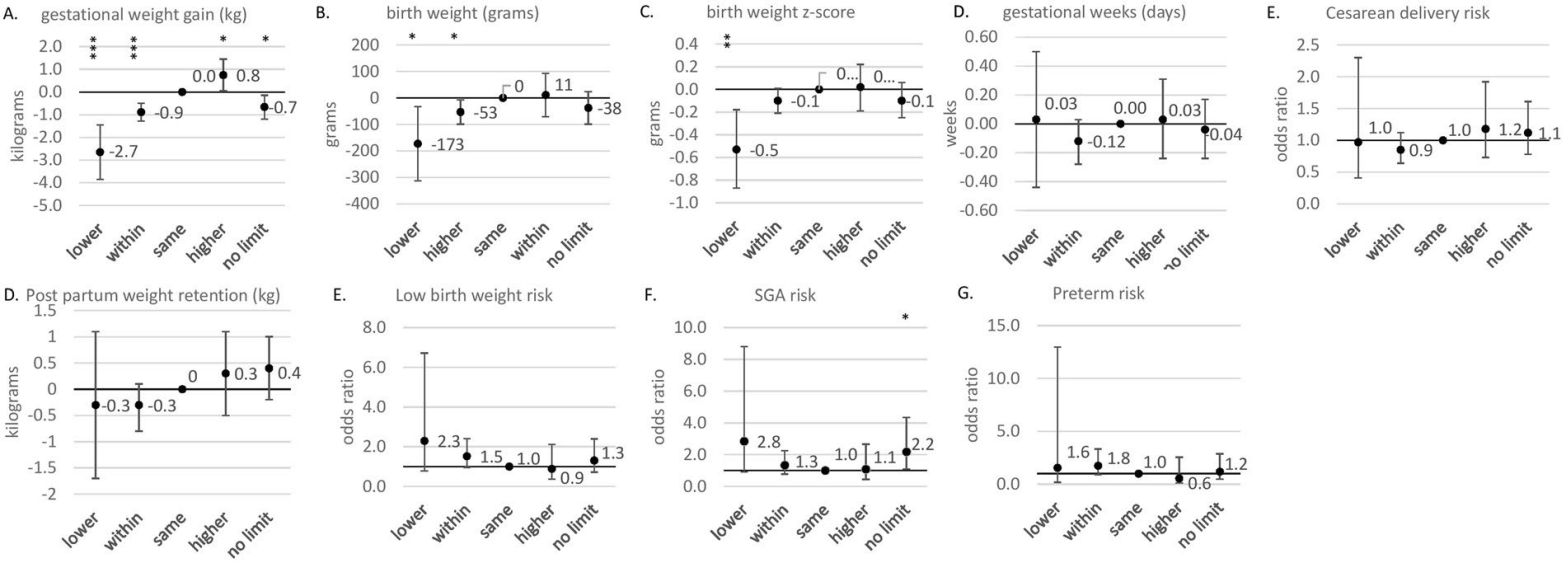
Association between birth outcomes and the upper limit of ideal gestational weight gain ranges among 1,691 women. *p* < 0.05; **p < 0.01; ***p < 0.001. Dots represent effect estimates and bars represent 95% confidence intervals. Estimates on post-partum weight retention were calculated using weight change from self-reported weight before pregnancy to self-reported weight at 12 months post-partum. The sample size for this analysis was N = 1,032. All models were adjusted for maternal age, parity, height, BMI, history of previous preterm delivery, maternal education, family income, smoking status during pregnancy, and the sex of infant. Weight gain at 40 weeks was calculated from all antenatal weight gain measurements available assuming linear increase in gestational weight in the third trimester.

Figure 1 shows the association between women’s perceived ideal GWG categories and their pregnancy outcomes after adjusting for maternal age, parity, height, pre-pregnancy BMI, history of previous preterm delivery, maternal education, family income, smoking status during pregnancy, and the sex of the infant. Estimated values for birth outcomes and risk of adverse outcomes by perceived ideal GWG category are shown in Tables 3 and 4, respectively. Women who chose an upper limit below the government recommendations gained 2.6 kg [95% confidence interval (CI), 1.4–3.8] less, had infants with a birthweight 173 grams lower (95% CI, 33–312), and a lower average birthweight z-score 0.5 (95% CI, 0.2–0.9) than women who reported an upper limit congruent with the guideline recommendations (12 kg). The risk of low birth weight [aOR: 2.3 (95% CI, 0.8–6.7)], SGA [aOR 2.8 (95% CI, 0.9–8.8)] and preterm delivery [aOR 1.6 (0.2–13.0)], as well as risk of cesarean delivery rate [aOR: 1.0 (95% CI, 0.4–2.3)] and average PPWR [aOR: −0.3 (95% CI, −1.7–1.1)kg] were not statistically different than in women who reported an upper limit congruent with the guideline recommendations (12 kg). Similarly, those who chose an upper limit within the guideline range but lower than 12 kg gained 0.9 kg less (95% CI, −1.4–3.8) and had an infant with a birthweight 53 grams lower (95% CI, 15–108) on average. The risk of low birth weight [aOR 1.5 (95% CI, 1.0–2.4)], SGA [1.3 (95% CI 0.8–2.3)], preterm delivery [1.8 (95% CI 0.8–2.3)] and cesarean delivery [0.8 (95% CI, 0.6–1.1)] and the average PPWR [−0.3 (95% CI, −0.8–0.1) kg] were not statistically different from the values of women who reported an upper limit congruent with the guideline recommendation (12 kg).

**Table 3:** Association between birth outcomes and the upper limit of ideal gestational weight gain ranges among 1,691 women.

Women’s perceived upper limit of ideal GWG categorized according to the Ministry of Health, Labour and Welfare guidelines^e^	Weight gain at 40 weeks^+c^ (kg) [95% CI^b^]	Birth weight (grams) [95% CI^b^]	Birth weight z-score [95% CI^b^]	Gestational length (weeks) [95% CI^b^]	Post-partum weight retention^++d^ (kg) [95% CI^b^]
Lower than guidelines	−2.6***^a^	−173*^a^	−0.5**^a^	0.0	−0.3
	[−3.8, −1.4]	[−312, −33]	[−0.9, −0.2]	[−0.4, 0.5]	[−1.7, 1.1]
Within guidelines but lower than upper limit (12 kg)	−0.9***^a^	−53*^a^	−0.1	−0.1	−0.3
	[−1.3, −0.5]	[−99, −7]	[−0.2, 0.0]	[−0.3, 0.0]	[−0.8, 0.1]
Same as upper limit (12 kg)	0	0	0	0	0
	Reference	Reference	reference	Reference	Reference
Higher than guidelines	0.8*^a^	11	0.0	0.0	0.3
	[0.1, 1.5]	[−70, 93]	[−0.2, 0.2]	[−0.2, 0.3]	[−0.5, 1.1]
No limit/not sure	−0.7*^a^	−38	−0.1	−0.0	0.4
	[−1.2, −0.1]	[−99, 24]	[−0.2, 0.1]	[−0.2, 0.2]	[−0.2, 1.0]

^a^*p < 0.05; **p < 0.01; ***p < 0.001.

^b^95% CI: 95% confidence interval.

^c+^Weight gain at 40 weeks was calculated from all antenatal weight gain measurements available assuming linear increase in gestational weight in the third trimester.

^d++^Calculated from self-reported weight at 12 months post-partum. Sample size for this analysis was N = 1,032.

^e^All models adjusted for maternal age, parity, height, BMI, history of previous preterm delivery, maternal education, family income, smoking status during pregnancy, and sex of infant.

**Table 4:** Association between risk of adverse outcomes and the upper limit of ideal gestational weight gain ranges among 1,691 women.

Women’s perceived upper limit of ideal GWG categorized according to the Ministry of Health, Labour and Welfare guidelines^e^	Low birth weight OR^b^ [95% CI]^c^	Small for gestational age OR^b^ [95% CI]^c^	Preterm delivery OR^b^ [95% CI]^c^	Cesarean delivery OR^b^ [95% CI]^c^
Lower than guidelines	2.3	2.8	1.6	1.0
	[0.8, 6.7]	[0.9, 8.8]	[0.2, 13.0]	[0.4, 2.3]
Within guidelines but lower than upper limit (12 kg)^d^	1.5	1.3	1.7	0.8
	[1.0, 2.4]	[0.8, 2.2]	[0.9, 3.3]	[0.6, 1.1]
Same as upper limit (12 kg)^d^	1	1	1	1
	reference	reference	reference	reference
Higher than guidelines	0.9	1.1	0.6	1.2
	[0.4, 2.1]	[0.4, 2.7]	[0.1, 2.6]	[0.7, 1.9]
No limit/not sure	1.3	2.2*	1.2	1.1
	[0.7, 2.4]	[1.1, 4.3]	[0.5, 2.9]	[0.8, 1.6]

^a^*p < 0.05; **p < 0.01; ***p < 0.001.

^b^OR: odds ratio.

^c^95% CI: 95% confidence interval.

^d^Ministry of Health, Labor and Welfare recommends gestational weight gain to be 9–12 kg if pre-pregnancy BMI is <18.5 kg/m^2^, and 7–12 kg if pre-pregnancy BMI is 18.5–25 kg/m^2^.

^e^Adjusted for maternal age, parity, height, BMI, history of previous preterm delivery, maternal education, family income, smoking status during pregnancy, and sex of the infant.

However, women who set an upper limit higher than the recommended value gained 0.8 kg (95% CI, 0.1–1.5) more than women whose perceived ideal GWG was congruent with the upper limit of the guideline recommendations, but no significant difference in the risk of low birth weight [aOR: 0.9 (95% CI, 0.4–2.1)], SGA [aOR: 1.1 (95% CI, 0.4–2.7)] or risk of preterm delivery [aOR: 0.6 (95% CI, 0.1–2.6)] was observed. Women who reported “no limit/not sure” for their upper limit gained 0.7 kg (95% CI, 0.1–1.2) less and did not have a significantly higher risk of low birth weight, SGA birth or preterm delivery than women who adopted the guideline value. Women in these two strata did not differ from those who had a perceived ideal GWG congruent with the guideline value in terms of PPWR or the risk of cesarean delivery.

Sensitivity analysis adjusted for the range of perceived ideal GWG and conducted in a population limited to women who reported both a lower and upper limit for the perceived ideal GWG range showed similar results (Appendix Tables 1 and 2). The crude associations between the category of the upper limit of women’s perceived ideal GWG and pregnancy outcomes are shown in Appendix Table 3.

## Discussion

We found that women who thought that the upper limit of the current Ministry of Health, Labour and Welfare guidelines on GWG was higher than their ideal gained significantly less weight. Although such women believed more strongly that avoiding excessive weight gain would ease their delivery and lead to better health, they did not show a reduced risk of cesarean delivery or lower PPWR. We also found that their infants had significantly lower birthweight although they did not have a significantly higher risk of low birthweight, SGA or preterm birth.

In our study, we found a strong association between the upper limit of perceived ideal GWG (obtained at 26 to 38 weeks gestation) and actual GWG (calculated from weight at delivery and pre-pregnancy weight). One small, recent study of 166 women in the United States^10^ investigated the association between perceived ideal and actual GWG. However, the study reported neither the association between perceived ideal GWG and pregnancy outcomes nor the women’s reasons for having their perceived ideal, as our study did. Even when provided with similar information from the guidelines, not all women followed the recommendations^17^. Our findings suggest that women’s perceived ideal GWG closely correlated with whether they thought that avoiding excess gain would help avoid stretch marks, reduce weight retention, and ease their delivery. Notably, the majority of women were well-educated, and all were given information regarding the current GWG recommendations at their antenatal visits. Thus the range of perceived ideal GWG indicated in the women’s responses did not substantially differ from the government recommendations (only 6% chose an upper limit higher than the recommended range while only 12% chose a lower limit below the recommended range).

Furthermore we found that such perceived ideals may influence birthweight by altering actual GWG. This is the first study describing pregnant women’s notions of perceived ideal GWG and their relationship to pregnancy outcomes. In our cohort, women who set a upper limits for GWG as “≦9 kg” delivered an infant that was more than 100 gm smaller (and although not statistically significant, had twice the risk of low birth weight as well) compared to women who reported 12 kg (the upper limit in the Japanese recommendations for normal and underweight women) as the upper limit of their perceived ideal GWG. Our findings suggest that women’s attitudes towards weight gain may influence birthweight by changing the actual gestational weight gain.

The current recommendation includes a 3–5 kg range for adequate GWG (e.g. 7–12 kg for underweight women with BMI under 18.5 kg/m^2^ and 9–12 kg for normal women with a BMI 18.5–25.0 kg/m^2^), leaving room for some variation in the perceived ideal GWG even while staying within the guideline-recommended range. The majority of women in our study indicated that they hoped to keep their GWG below the upper limit recommended by the government guidelines, but in our study these women had infants with a lower birthweight. Our findings suggest that ‘personal’ notions of perceived ideal GWG that vary by several kilograms can have an impact on birthweight.

In our study 80% of women responded that they considered avoiding excessive weight gain was important “for ease of delivery and/or her health”. However, neither risk of cesarean delivery nor post-partum body weight retention was lower among women who chose a lower weight limit for their perceived ideal GWG. Studies conducted in other populations conducted in overweight as well as normal and underweight women have shown that excess GWG increases the risk of cesarean delivery and post-partum body weight retention. One possible reason we failed to detect an association is that the current Japanese Ministry of Health, Labour and Welfare guidelines are already quite strict^15^; thus there is no benefit in further restriction. It is even possible that further restriction may lead to suboptimal growth of the fetus, leading to a possible increase in risk of cesarean section^18^. Indeed recent research suggested that weight gain recommended by the current national guidelines may be lower than what provides most optimal pregnancy outcomes^15^.

So what does our study suggest? We observed that among normal and underweight women, a majority reported that their perceived ideal GWG was lower than current Japanese recommendation by Ministry of Health, Labour and Welfare guidelines. While they considered avoiding excess weight gain as important for easier delivery and/or their health, we failed to observe any reduction in cesarean delivery rates or post-partum weight retention among these women. On the other hand, we observed that actual GWG correlated with the upper limit of the perceived ideal GWG. As many studies show that actual GWG is associated with many birth outcomes^15,19,20^, we believe our study suggests that women may not be aware of the true consequences of excessive or inadequate GWG. Thus, providing more detailed antenatal education on the influence of GWG on pregnancy outcomes may help women achieve adequate gestational weight gain and thus better birth outcomes.

Furthermore, our findings may help understand the increased prevalence of low birthweight births in Japan that has been observed over the past two decades. A high proportion (54%) of women in our study answered that their perceived ideal GWG was below guideline recommendations. Even though there was no increase in risk of low birthweight births due to lower perceived ideal GWG, we cannot deny the possibility that changes in maternal values (such as an increase in women who consider that weight gain below the guideline recommendations would help ease delivery) leading to declines in perceived ideal GWG contributed to the increase in cases of low birthweight births.

There are several limitations to our study. First, as the women were surveyed using an unvalidated questionnaire, social desirability bias may have led them to give a GWG range closer to the Japanese Ministry of Health, Labour and Welfare recommendation. However, we found that perceived ideal GWG and actual GWG were very highly correlated, suggesting that the effect of such a bias is likely to be small. Second, our study was based on a relatively small sample, and adjusting for numerous confounders may have led to model over-fitting; thus future studies should be aimed at determining whether our findings, especially those relating to preterm birth, are replicable and consistent. Third, although all the mothers in our study received antenatal education at the same institution based on the Ministry of Health, Labour and Welfare guidelines on GWG, the quantity and quality of the education may have varied according to the heath care provider.

Several limitations are also inherent in our study design. Our study used the ideal upper limit of GWG as the main measure of the perceived ideal GWG due to the nature of our questionnaire design (women were allowed to leave the lower limit blank). Although we believe the upper limit of GWG is by itself a meaningful indicator as women would be more conscious of adopting a weight gain target below rather than above a given value, self-restriction of weight gain should be measured more rigorously in future studies. Another limitation is that the reporting timing was during mid- to late- pregnancy (mean 31.0 weeks); thus we were unable to rule out the possibility that women with a lower GWG had reported a lower GWG as “ideal” to justify their actual GWG. While our finding that perceived ideal GWG was associated with maternal values (such as the notion of the importance of not gaining too much weight during pregnancy) suggests that such reverse causation does not explain all of our observed associations, future studies aiming to obtain information about perceived ideal GWG at an earlier stage of pregnancy, or even better, repeatedly (to check for the influence of education on maternal ideals) are desired. Last but not least, maternal values may differ according to maternal and cultural background; thus research on other populations is needed. It is important to note that we were not able to analyze overweight/obese women for whom there are no Japanese GWG recommendations to serve as a reference.

In conclusion, we found that women who reported an upper limit for perceived ideal GWG that was lower than the current Ministry of Health, Labour and Welfare guidelines gained less actual GWG than women who reported an upper limit congruent with the guidelines; however they did not show a reduced risk of cesarean delivery or lower PPWR even though they believed that avoiding excessive weight gain would ease delivery and lead to better health. In addition to educating women about the current GWG guidelines, providing more detailed antenatal education on the influence of GWG on the mother and the child may help improve pregnancy outcomes.

## Electronic supplementary material

Supplementary Information
