# Supplementary material for: Association between women’s perceived ideal gestational weight gain during pregnancy and pregnancy outcomes
Source: Sci Rep. 2018 Aug 1;8:11574. doi: 10.1038/s41598-018-29936-z (PMC6070475; doi:10.1038/s41598-018-29936-z)

Supplementary Information for Ogawa K, Morisaki N, Sago H, Fujiwara T, Horikawa R. Association between women's perceived ideal gestational weight gain during pregnancy and pregnancy outcomes.:

Appendix Table 1-3

Appendix Figure 1-2

Appendix Table 1. Association between pregnancy outcomes and the upper limit of ideal gestational weight gain ranges among 1,240 women

	Weight gain at 40 weeks (kg) [95% CI]	Birth weight (grams) [95% CI]	Birth weight z-score [95% CI]	Gestational length (weeks) [95% CI]	Post-partum weight retention+(kg) [95% CI]
Lower than the guideline recommendation	-2.8*** [-4.1 - -1.5]	-160* [-313 - -7]	-0.6** [-0.9 - -0.2]	0.1 [-0.4 - 0.7]	-0.5 [-2.0, 0.9]
Within the recommended range but lower than the upper limit	-1.1*** [-1.6 - -0.7]	-66* [-120 - -11]	-0.1 [-0.2 - 0.0]	-0.2 [-0.4 - 0.0]	-0.5 [-1.0, 0.0]
Same as the guideline recommendation (12kg)	0 Reference	0 Reference	0 reference	0 Reference	0 Reference
Higher than the guideline recommendation (12kg<)	1.1** [0.3 - 1.9]	17 [-80 - 113]	-0.0 [-0.2 - 0.2]	0.1 [-0.2 - 0.4]	0.5 [-0.4, 1.4]
No limit/not sure	NA	NA	NA	NA	NA

a. * $p<0.05$; ** $p<0.01$; *** $p<0.001$

b. 95%CI: 95% confidence interval

c. +: Calculated from self-reported weight at 12 months post-partum. N=759.

d. Ministry of Health, Labour and Welfare recommends gestational weight gain to be 9–12 kg if pre-pregnancy BMI is <18.5kg/m^2, and 7–12kg if pre-pregnancy BMI is 18.5-25 kg/m^2.

e. All models adjusted for maternal age, parity, height, BMI, history of previous preterm delivery, maternal education, family income, smoking status during pregnancy, sex of infant, as well as difference between upper and lower limits of reported ideal gestational weight gain.

f. Weight gain at 40 weeks was calculated from all antenatal weight gain measurements available assuming linear increase in gestational weight in the third trimester

Appendix Table 2. Association between risk of adverse outcomes and the upper limit of ideal gestational weight gain ranges among 1,240 women

	Low birth weight OR[95% CI]	Small for gestational age OR[95% CI]	Preterm delivery OR[95% CI]	Cesarean delivery OR[95% CI]
Lower than the guideline recommendation	1.5 [0.5 - 5.2]	3.1 [1.0 - 9.8]	NE	1.1 [0.5 - 2.7]
Within the recommended range but lower than the upper limit	1.3 [0.8 - 2.3]	1.3 [0.7 - 2.3]	1.7 [0.9 - 3.6]	0.9 [0.6 - 1.2]
Same as the guideline recommendation (12kg)	1 reference	1 reference	1 reference	1 reference
Higher than the guideline recommendation (12kg<)	0.8 [0.3 - 2.4]	0.9 [0.3 - 2.6]	0.6 [0.1 - 3.1]	1.5 [0.8 - 2.6]
No limit/not sure	NA	NA	NA	NA

a. * *p*<0.05; ** *p*<0.01; *** *p*<0.001

b. OR: odds ratio

c. 95%CI: 95%confidence interval

d. Ministry of Health, Labour and Welfare recommends gestational weight gain to be 9–12 kg if pre-pregnancy BMI is <18.5kg/m^2, and 7–12kg if pre-pregnancy BMI is 18.5-25 kg/m^2.

e. All models adjusted for maternal age, parity, height, BMI, history of previous preterm delivery, maternal education, family income, smoking status during pregnancy, sex of infant, as well as difference between upper and lower limit of reported ideal gestational weight gain.

Appendix Table 3. Crude association between pregnancy outcomes and the upper limit of ideal gestational weight gain ranges among 1,691 women

	Weight gain at 40 weeks (kg) [95% CI]	Birth weight (grams) [95% CI]	Birth weight z-score [95% CI]	Gestational length (weeks) [95% CI]	Post-partum weight retention^{+}(kg) [95% CI]
Lower than the guideline recommendation	-2.7*** [-3.9 - -1.5]	-214** [-358 - -70]	-0.7*** [-1.1 - -0.4]	-0.1 [-0.6 - 0.4]	0.3 [-1.1 – 1.6]
Within the recommended range but lower than the upper limit	-0.9*** [-1.3 - -0.5]	-47* [-95 - -0]	-0.1 [-0.2 - 0.0]	-0.2** [-0.4 - -0.1]	-0.4 [-0.8, 0.1]
Same as the guideline recommendation (12kg)	1 Reference	1 Reference	1 Reference	1 Reference	1 Reference
Higher than the guideline recommendation (12kg<)	0.9* [0.2 - 1.6]	9.4 [-75 - 94]	-0.0 [-0.2 - 0.2]	-0.1 [-0.4 - 0.2]	0.5 [-0.3 – 1.3]
No limit/not sure	-0.6* [-1.2 - -0.1]	-35.3 [-99 - 29]	-0.1 [-0.2 - 0.1]	-0.1 [-0.3 - 0.1]	0.4 [-0.2 – 1.1]

	Low birth weight OR[95% CI]	Small for gestational age OR[95% CI]	Preterm delivery OR[95% CI]	Cesarean delivery OR[95% CI]
Lower than the guideline recommendation	2.5 [0.9 - 7.0]	3.1* [1.1 - 8.7]	1.0 [0.1 - 7.7]	0.9 [0.4 - 2.0]
Within the recommended range but lower than the upper limit	1.5 [1.0 - 2.4]	1.3 [0.8 - 2.1]	1.8 [1.0 - 3.4]	0.9 [0.7 - 1.2]
Same as the guideline recommendation (12kg)	1 Reference	1 Reference	1 Reference	1 Reference
Higher than the guideline recommendation (12kg<)	0.9 [0.4 - 2.2]	1.1 [0.5 - 2.7]	0.6 [0.1 - 2.6]	1.2 [0.7 - 1.8]
No limit/not sure	1.4 [0.8 - 2.4]	2.0* [1.1 - 3.6]	1.2 [0.5 - 2.8]	1.2 [0.8 - 1.7]

a. * p<0.05; ** p<0.01; *** p<0.001
b. OR odds ratio
c. $^{+}$Calculated from self-reported weight at 12 months post-partum. N=1032.
d. Weight gain at 40 weeks was calculated from all antenatal weight gain measurements available assuming linear increase in gestational weight in the third trimester

Appendix Figure 1: Original Questionnaire

1. Questionnaire

Q1: How much weight do you think is appropriate for yourself to gain during pregnancy?

1: I don't know 2: ( ) to ( ) kg 3: ( ) kg or under

Q2: Do you think it is important not to gain too much weight during pregnancy?

1: very much so 2: somewhat so 3. not sure 4.not much 5.not at all

For those who answered "1: very much so" "2: somewhat so" please answer the following question.

Q3: Why do you think so? Choose all that apply.

1. to bear a healthy child 2. for ease of delivery
3. to get my body back into shape quickly after delivery 4. to avoid stretch marks
6. because my doctor/nurse/midwife said so 7. because my family/friends said so
7. no particular reason 8. to avoid lifestyle-related diseases later in life

Appendix Figure 2: Scatterplot of the upper limit and lower limit of ideal gestational weight gain among 1,240 women

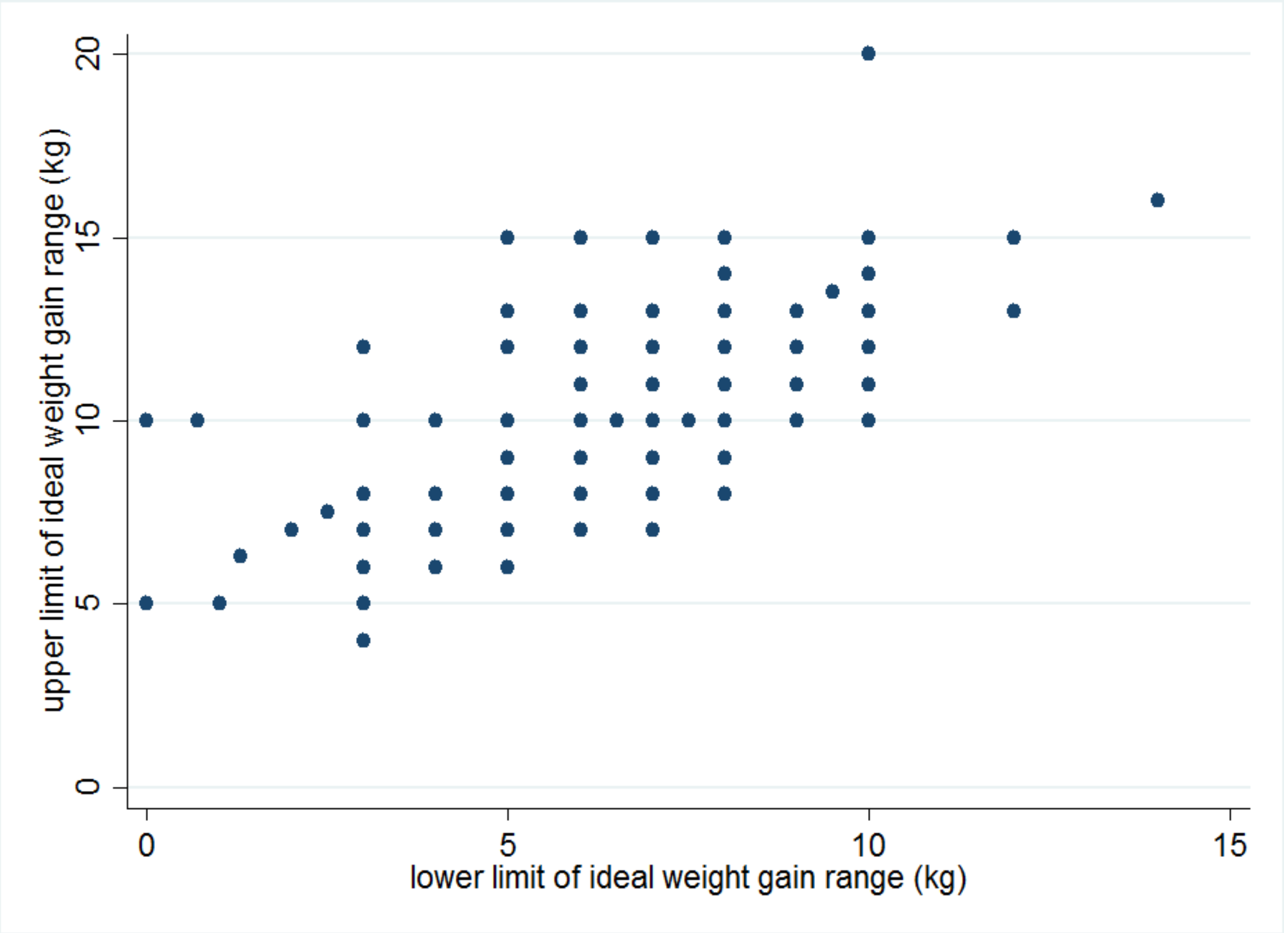

Supplement: Supplementary file 1 — Supplementary Information [file 41598_2018_29936_MOESM1_ESM.pdf]
